# Apelin and its ratio to lipid factors are associated with cardiovascular diseases: A systematic review and meta-analysis

**DOI:** 10.1371/journal.pone.0271899

**Published:** 2022-08-01

**Authors:** Hamed Akbari, Mahnaz Hosseini-Bensenjan, Sarvenaz Salahi, Fatemeh Moazzen, Hamid Aria, Alireza Manafi, Saeed Hosseini, Maryam Niknam, Gholamreza Asadikaram

**Affiliations:** 1 Department of Biochemistry, School of Medicine, Kerman University of Medical Sciences, Kerman, Iran; 2 Student Research Committee, School of Medicine, Kerman University of Medical Sciences, Kerman, Iran; 3 Hematology Research Center, Shiraz University of Medical Sciences, Shiraz, Iran; 4 Minimally Invasive Surgery Research Center, Iran University of Medical Sciences, Tehran, Iran; 5 Department of Hematology, Faculty of Allied Medicine, Bushehr University of Medical Sciences, Bushehr, Iran; 6 Department of Immunology, School of Medicine, Isfahan University of Medical Sciences, Isfahan, Iran; 7 Brigham and Women`s Hospital, Harvard Medical School, Boston, Massachusetts, United States of America; 8 Center for Healthcare Data Modeling, Department of Biostatistics and Epidemiology, School of Public Health, Shahid Sadoughi University of Medical Sciences, Yazd, Iran; 9 Department of Epidemiology, School of Public Health, Iran University of Medical Sciences, Tehran, Iran; 10 Department of Biochemistry, School of Medicine, Shiraz University of Medical Sciences, Shiraz, Iran; 11 Neuroscience Research Center, Institute of Neuropharmacology, Kerman University of Medical Sciences, Kerman, Iran; The University of Mississippi Medical Center, UNITED STATES

## Abstract

**Background:**

The present systematic review and meta-analysis aimed to ascertain if the circulating levels of apelin, as an important regulator of the cardiovascular homeostasis, differ in patients with cardiovascular diseases (CVDs) and controls.

**Methods:**

A comprehensive search was performed in electronic databases including PubMed, Scopus, EMBASE, and Web of Science to identify the studies addressing apelin in CVD up to April 5, 2021. Due to the presence of different units to measure the circulating levels of apelin across the included studies, they expressed the standardized mean difference (SMD) and their 95% confidence interval (CI) as summary effect size. A random-effects model comprising DerSimonian and Laird method was used to pool SMDs.

**Results:**

Twenty-four articles (30 studies) comprised of 1793 cases and 1416 controls were included. Pooled results obtained through random-effects model indicated that apelin concentrations in the cases’ blood samples were significantly lower than those of the control groups (SMD = -0.72, 95% CI: -1.25, -0.18, P = 0.009; I^2^ = 97.3%, P<0.001). New combined biomarkers showed a significant decrease in SMD of apelin/high-density lipoprotein cholesterol (apelin/HDL-C) ratio [-5.17; 95% CI, -8.72, -1.63, P = 0.000; I^2^ = 99.0%], apelin/low-density lipoprotein cholesterol (apelin/LDL-C) ratio [-4.31; 95% CI, -6.08, -2.55, P = 0.000; I^2^ = 98.0%] and apelin/total cholesterol (apelin/TC) ratio [-17.30; 95% CI, -22.85, -11.76, P = 0.000; I^2^ = 99.1%]. However, no significant differences were found in the SMD of apelin/triacylglycerol (apelin/TG) ratio in cases with CVDs compared to the control group [-2.96; 95% CI, -7.41, 1.49, P = 0.000; I^2^ = 99.2%].

**Conclusion:**

The association of apelin with CVDs is different based on the region and disease subtypes. These findings account for the possible usefulness of apelin as an additional biomarker in the diagnosis of CVD in diabetic patients and in the diagnosis of patients with CAD. Moreover, apelin/HDL-c, apelin/LDL-c, and apelin/TC ratios could be offered as diagnostic markers for CVD.

## Introduction

Cardiovascular diseases (CVDs) are one of the main life-threatening diseases with high prevalence [1, 2]. Globally, CVDs are responsible for 31% of mortality, the majority of this in the form of coronary heart disease (CHD) and cerebrovascular accident. It has been recommended that improvement of the disease risk factors, including dyslipidemia, smoking, hypertension, diabetes, and abdominal obesity is important in CVD prevention [3–5]. As an active endocrine organ, white adipose tissue plays crucial roles in obesity-related CVD including the secretion of adipokines that affect the whole-body homeostasis [6]. One of these adipokines, apelin, is one of the most potent endogenous positive inotropic compounds yet identified [7]. It is widely distributed in the heart and may act as an important regulator of the cardiovascular homeostasis [8].

Although previouse reports confirm that apelin is involved in cardiovascular function, there is controversy about its causative association with CVDs [9]. In addition, the acceleration of CVD prevention via early diagnosis and treatment of risk factors is still a ctitical issue [10]. It has been revealed that the role of apelin in the cardiovascular field is widespread. Although it remains to be seen whether apelin will translate into a therapeutic target in the future, the results of previous studies confirm the importance of further investigation. More functional studies are required to determine the exact role of apelin/APJ in cardiovascular regulation. The present systematic review and meta-analysis aimed to summarize the available data about the circulating levels of apelin as a possible regulator of the cardiovascular homeostasis in patients with CVDs. Considering the acute influences of apelin on lipid metabolism and its correlation with total cholesterol (TC), triacylglycerol (TG), low-density lipoprotein cholesterol (LDL-C), very low density lipoprotein cholesterol (VLDL-C), and high-density lipoprotein cholesterol (HDL-C), as crucial factors for the development of atherosclerotic plaque, we also calculated the ratios of apelin to lipid profile levels for cases and controls. This comprehensive data would further enhance our knowledge of the function of apelin and its receptor in CVDs and help us to assess this receptor system as a future drug target.

## Methods

PRISMA guideline (the Preferred Reporting Items for Systematic Reviews and Meta-analysis) was used to design and perform the current meta-analysis (S1 Checklist) [11]. Moreover, no external board review was conducted before doing this study.

### Search strategy

Two independent authors (MH-B and SS) performed a comprehensive search to identify the relevant published English language studies through inception up to April 5, 2021. The controversies were checked by a third expert person (MN). Electronic databases including PubMed, Scopus, EMBASE, and Web of Science were searched by using the following MeSH (Medical Subject Heading) terms and text keywords: (Apelin) AND ("Coronary Artery Disease" OR "Myocardial Ischemia" OR "Acute Coronary Syndrome" OR "Angina, Stable" OR "Angina, Unstable" OR "Coronary Disease" OR "Coronary Stenosis" OR "Myocardial Infarction" OR "Non-ST Elevated Myocardial Infarction" OR "ST Elevation Myocardial Infarction"). We also conducted a manual search in the reference list’s included articles and pervious relevant reviews to increase the sensitivity of searches to find additional articles. Pubmed search strategy is presented in Supp 2. a in S1 File.

### Study selection

English published articles that met the inclusion criteria were selected. Inclusion criteria were as follows: original observational studies including cross-sectional, case-control, and clinical cohort studies; and studies providing detailed information regarding blood circulating levels of apelin in patients diagnosed with heart diseases such as coronary artery disease (CAD), myocardial infarction (MI), congenital heart disease (CHD), congestive heart failure (CHF), heart failure (HF), atrial fibrillation (AF), ischemic heart disease (IHD), hypertensive heart disease (HHD) and controls (participants without heart diseases and other chronic/metabolic diseases). Studies were also excluded if they investigated animal models, using animal models, tissue-based cultures, cell cultures, and mRNA expression; and case reports, conference abstracts, comments, review articles, editorials, and articles without insufficient data. The title and the abstract of each article were reviewed by two independent investigators (FM and HA). Following this initial screening step, potential articles were included in our full-text review process. Any existing discrepancies were resolved by consensus or consultation with a third author (AM).

### Data extraction and quality assessment

Following the identification of eligible studies, data extraction was done by three individual authors (HA, SH, and HA) using pre-designed data collection sheets in Excel. The first author’s name, publication year, geographical region, age, study design, study sample size, and apelin concentration in CVD patients and controls (means ± standard deviation (SD)) were collected. The quality of the study was evaluated using the Newcastle-Ottawa Quality Assessment Scale (NOS) [12], which involved evaluation of study design and analysis, selection bias, measurements of exposure and outcome, and generalizability of results. The NOS tool includes nine items with scores ranging from 0 to 9. Based on the type of study, quality scores ≥ 5 in cross-sectional designs and ≥ 7 in case-control or cohort designs represented good quality.

### Statistical analysis

All meta-analyses were performed using STATA 11.0 (STATA Corp, College Station, TX). Given the different units of blood circulation levels of apelin across the included studies, they were expressed as standardized mean difference (SMD) and their 95% confidence interval (CI) was presented as summary effect size using Hedges and Olkin standard error. Moreover, with respect to the bias-correlation factor in effect size, an exact computation was used. For calculating the ratios of apelin to lipids, we used the following formula: To calculate the apelin/HDL-c ratio: mean aeplin / mean HDL-c in both groups (cases and controls). Then, the SD ratio using the following formula: (mean_apelin_^2^ / mean_HDL_^2^) × [(SD_apelin_^2^ / mean_apelin_^2^)– 2 (R× SD_apelin_ × SD_HDL_ / meanapelin × mean_HDL_) + (SD_HDL_^2^ / mean_HDL_^2^)] [13, 14]. The correlation coefficients (Rs) for HDL-c, LDL-c, TC, and TG were included based on the studies conducted by Bilik et al. and Sun et al. [15, 16]. The selected studies were combined using the DerSimonian and Laird random effects model or the inverse variance fixed-effect model, depending on the existence of significant heterogeneity (I^2^ ≥ 50% with P < 0.05) [17]. Subgroup analyses were conducted on study design, comorbidities of diabetes/MetS, body fluid, type of heart disease, and continent to explore the source of heterogeneity. Sensitivity analyses were performed as additional analyses using the leave-one-out method to examine the influence one by one study on the validity of the pooled SMDs. Egger’s test and Funnel plot were also applied to assess the potential publication bias.

## Results

### Literature search and study characteristics

**Fig 1:**
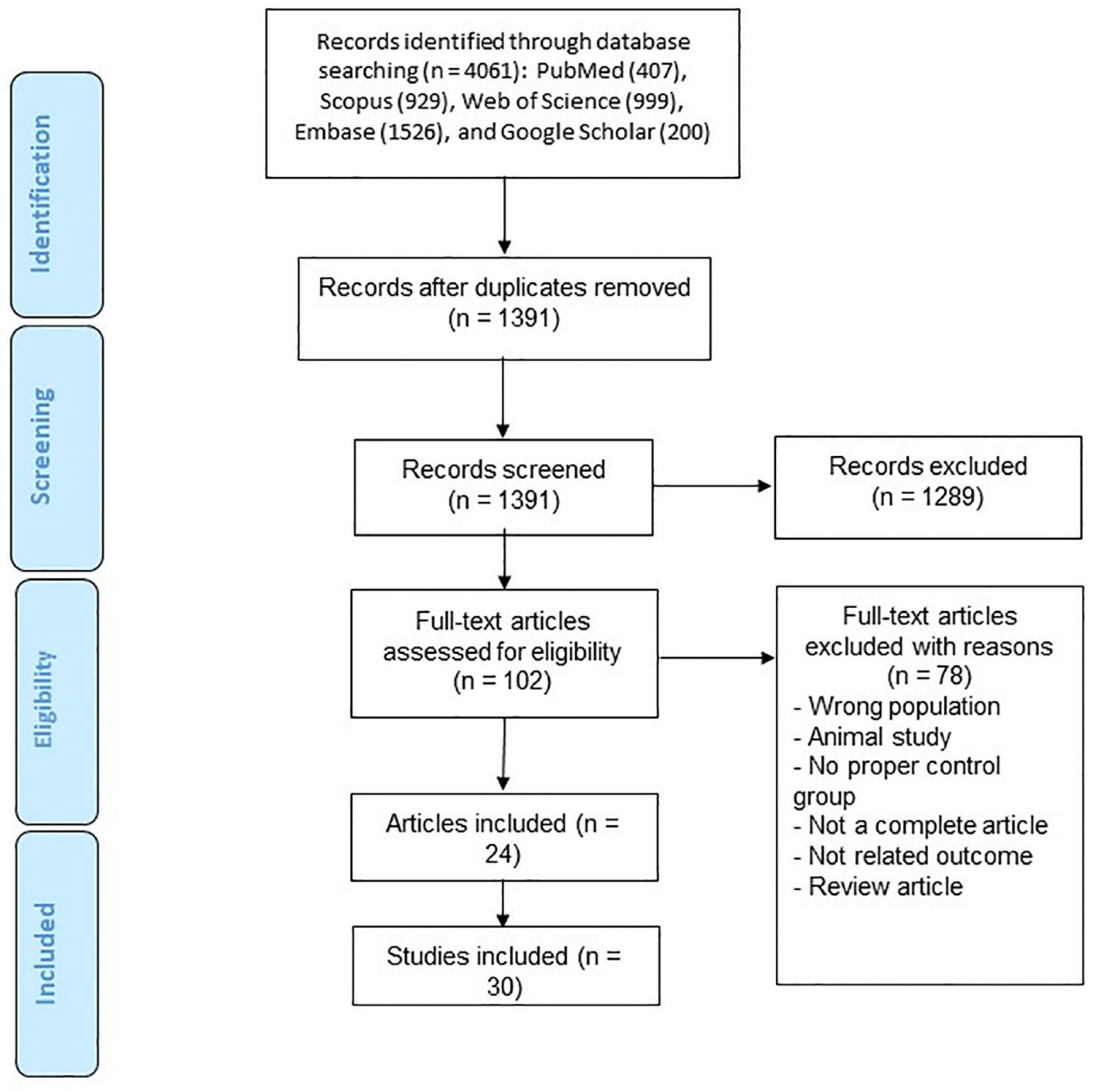
The flowchart of study identification and selection process.

Fig 1 demonstrates the step by step process of identification and selection of relevant studies with more detail. The primary systematic search resulted in the retrieval of 4061 records. Of these, 2670 were excluded as duplicates and 1391 remained as the screened records. Finally, 24 articles (30 studies) were included as the final records of the current meta-analysis [6, 15, 16, 18–38]. The included studies had been published between 2006 and 2020 and were comprised of 1793 cases and 1416 controls. The number of participants in each group varied from 8 to 202. Twelve studies reported data on CAD, and the remaining were based on other CVDs. Ten studies were performed in Asia, 13 in Europe, five in Africa, and two in America. Twenty-four, four, and two studies were conducted with case-control, cohort, cross-sectional design, respectively. The characteristics of selected studies are summarized in Table 1.

**Table 1:** Main characteristics of included studies.

Author	Year	Total sample size	Study design	Country	Mean age (control vs. case)	Gender male/female (control vs. case)	BMI (kg/m^2^) (control vs. case)	Cases	Controls	Comorbidities of diabetes/MetS	Name of comorbidities	Body fluid	Quality scores
Abdelaziz [18]	2015	60	Case-control	Egypt	56.05 ± 7.04	NR, 30/10	NR, 25.3	CAD	Healthy subjects	With	HT, DM	Plasma	High
Abd-Elbaky [19]	2016	160	Case-control	Egypt	38.6±4.2, 40.3±2.5	All men	21, 32.6	CVD	Healthy, non-obese controls	With	T2DM	Serum	Low
Akcılar [21]	2015	276	Case-control	Turkey	61.5 ± 10.75, 64.2 ± 11.94	64/54, 117/41	NR	CAD	Healthy subjects	Without	-	Plasma	Low
Basile [22]	2014	50	Case-control	Italy	80 ± 7.8,	Sex matched controls, 16/14	NR	CHF	Healthy subjects	Without	-	Serum	Low
Bilik [15]	2015	54	Case-control	Turkey	51.6 ± 8.8, 53.6 ± 8.1	18/10, 19/7	26.7, 28.1	CAE	Patients with normal coronary arteries	With	HT, DM	Plasma	High
Celik [23]	2016	76	Case-control	Turkey	53.33±40.69, 60±53.26	9/11, 25/31	NR	Right ventricular dysfunction	Healthy subjects	NR	Acute pulmonary embolism	Plasma	High
Chong [24]	2006	224	Cohort	United Kingdom	51.3 ± 9.2, 51.7 ± 11.6	16/6, 157/45	26.4, 28.8	CHF	No history of cardiac events	Without	Left ventricular systolic dysfunction	Plasma	High
El Amrousy [25]	2018	120	Case-control	Egypt	15.9± 7.8, 15.6±8.3	28/32, 28/32	NR	HF	Healthy children	Without	Congenital heart disease	Serum	High
Ellinor [26]	2006	146	Cohort	USA	54.3, 54.2	58/15, 58/15	NR	AF	Healthy subjects	NR	-	Plasma	High
Francia [27]	2007	22	Case-control	Italy	68±13	Sex matched controls, 9/5	NR	CHF	Healthy subjects	Without	-	Plasma	High
Gurger#a [28]	2014	59	Case-control	Turkey	40 ± 8, 45 ±7	43.3% Male, 39.5% Male	NR	Lone AF	Healthy subjects	With	DM, HT	Plasma	High
Gurger#b [28]	2014	59	Case-control	Turkey	40±8, 42±9	43.3% Male, 44.4% Male	NR	PSVT	Healthy subjects	With	DM, HT	Plasma	High
Hazbar [20]	2018	84	Case-control	Iraq	55.38±10.35, 57.78±9.85	20/4, 32/28	23.82, 26.56	CAD	Healthy subjects	Without	-	Plasma	High
Małyszko [39]	2006	81	Cross-sectional	Poland	56.10 ± 15.07, 63.42 ± 10.01	NR	24.2, 24.8	HD with CAD	HD without CAD	Without	Renal failure	Plasma	High
Miettinen [30]	2007	79	Case-control	Finland	61±11, 53±12	2/12, 50/15	NR	IDC	Healthy subjects	Without	-	Plasma	High
Motawi#a [6]	2018	60	Case-control	Egypt	54.6±3.1, 53.7±7.6	14/16, 23/22	21.8, 23.1	CAD	Healthy subjects	Without	-	Serum	High
Motawi#b [6]	2018	60	Case-control	Egypt	54.6±3.1, 55.3±6	14/16, 11/34	21.8, 32.5	CAD with diabetes and obesity	Healthy subjects	With	T2DM	Serum	High
Pang#a [31]	2015	44	Case-control	China	67.43 ± 7.43, 67.05 ± 12.51	19/21, 11/13	NR	HHD	Healthy subjects	With	DM, Hyperlipidemia, Atrial fibrillation, Renal impairments	Plasma	High
Pang#b [31]	2015	55	Case-control	China	67.43 ± 7.43, 68.05 ± 11.47	19/21, 17/18	NR	HHD+CAD	Healthy subjects	With	DM, Hyperlipidemia, Atrial fibrillation, Renal impairments-	Plasma	High
Rachwalik [32]	2011	33	Case-control	Poland	51± 15.56, 52 ± 17.79	9/7, 11/6	23.9, 28.2	CAD	Healthy subjects	With	DM	Serum	High
Şimşek [33]	2019	120	Cross-sectional	Turkey	38.6± 9.9, 39 ± 14	40/18, 45/17	26.0, 26.5	BAV	Healthy subjects	Without	-	Serum	High
Sun#a [16]	2020	75	Case-control	China	58.06 ± 9.51, 62.08 ±8.29	15/35, 29/21	25.02, 25.5	CAD	Patients without CAD	With	DM, HT	Serum	High
Sun#b [16]	2020	65	Case-control	China	58.06 ± 9.51, 61.28 ±8.46	15/35, 26/14	25.02, 25.17	CAE	Patients without CAD	With	DM, HT	Serum	High
van Kimmenade [34]	2006	599	Cohort	US	56.9 ±16.3, 72.8±13.6	51% male, 51% male	NR	Acute HF	No Acute HF	With	DM, HT, Obstructive lung disease	Plasma	High
Velliou [35]	2020	100	Case-control	Greece	58.9 ± 10.7, 60.6 ± 12.1	28/22, 29/21	28.3, 28	AF	Non-AF	With	Obesity, MetS, DM, Dyslipidemia, HT	NR	High
Wang [36]	2018	61	Cohort	China	53.81±15.84, 58.50±7.56	23/13, 13/9	23.23, 23.1	ERAF	No ERAF	With	DM, HTN, AF	Serum	High
Wilson [37]	2014	20	Case-control	India	34 ± 10, 26 ± 6	7/3, 4/6	22.8, 22.2	Mitral Stenosis patients	Healthy subjects	Without	-	Plasma	High
Zhou#a [40]	2014	122	Case-control	China	56.9 ± 4.1, 55.7 ± 3.4	62.8% male,63.1% male	NR	STEMI patients	No coronary heart disease	Without	Hypertension, Hyperlipidemia, Cerebrovascular disease	Plasma	High
Zhou#b [40]	2014	119	Case-control	China	56.9 ± 4.1, 57.2 ± 2.5	62.8% male, 64.5% male	NR	Non-STEMI patients	No coronary heart disease	Without	Hypertension, Hyperlipidemia, Cerebrovascular disease	Plasma	High
Zhou#c [40]	2014	126	Case-control	China	56.9 ± 4.1, 56.3 ± 2.8	62.8% male, 61.8% male	NR	Stable angina patients	No coronary heart disease	Without	Hypertension, Hyperlipidemia, Cerebrovascular disease	Plasma	High

**Abbreviations**: CAD: coronary artery disease, CVD: cardiovascular disease, CHD: congenital heart disease, CHF: congestive heart failure, CAE: coronary artery ectesia, HF: heart failure, AF: atrial fibrillation, MI: myocardial infarction, DM: diabetes mellitus, PSVT: paroxysmal supraventricular tachycardia, AMI: acute myocardial infarction, IHD: ischemic heart disease, HD: hemodialysis, SAP: stable angina pectoris, UAP: unstable angina pectoris, IDC: idiopathic dilated cardiomyopathy, ADHF: acute decompensated heart failure, HHD: hypertensive heart disease, BAV: bicuspid aortic valve, ERAF: early recurrence of atrial fibrillation, ACS: acute coronary syndrome, STEMI: ST elevation myocardial infarction, Non-STEMI: non ST elevation myocardial infarction, NR: not reported, MetS: metabolic syndrome, HT: hypertension.

### Pooled effect of apelin and its ratios to lipid profile levels between cases and controls

**Fig 2:**
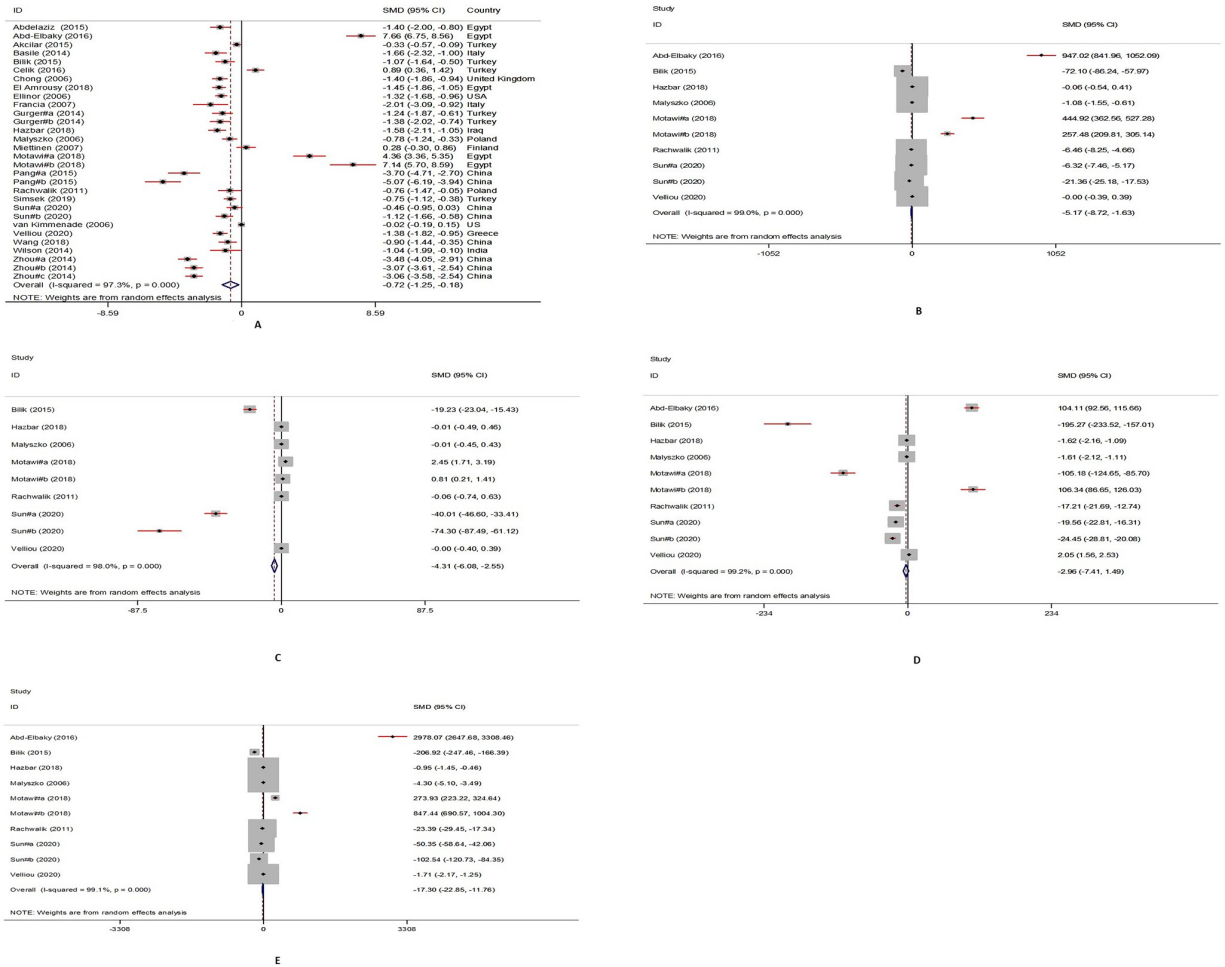
The forest plots of pooled estimates of standardized mean differences of circulating apelin (A), apelin/HDL-c (B), apelin/LDL-c (C), apelin/triglycerides (D), and apelin/total cholesterol (E) levels between cases with CVDs and controls.

Based on the 30 selected studies, the meta-analysis finding indicated that the blood apelin concentrations among cases were significantly lower than those of the control groups (SMD = -0.72, 95% CI: -1.25, -0.18, P = 0.009; I^2^ = 97.3%, P<0.001). Findings of new combined markers demonstrated a significant decrease in SMD of apelin/HDL-c ratio [-5.17; 95% CI, -8.72, -1.63, P = 0.000; I^2^ = 99.0%], apelin/LDL-c ratio [-4.31; 95% CI, -6.08, -2.55, P = 0.000; I^2^ = 98.0%] and apelin/TC ratio [-17.30; 95% CI, -22.85, -11.76, P = 0.000; I^2^ = 99.1%]. However, no significant differences were found in the SMD of the apelin/TG ratio in cases with CVDs compared to the control group [-2.96; 95% CI, -7.41, 1.49, P = 0.000; I2 = 99.2%]. Forest plots that indicated the pooled SMD and each study on the apelin and new combined markers concentrations between cases and controls are shown in Fig 2A–2E.

**Fig 3:**
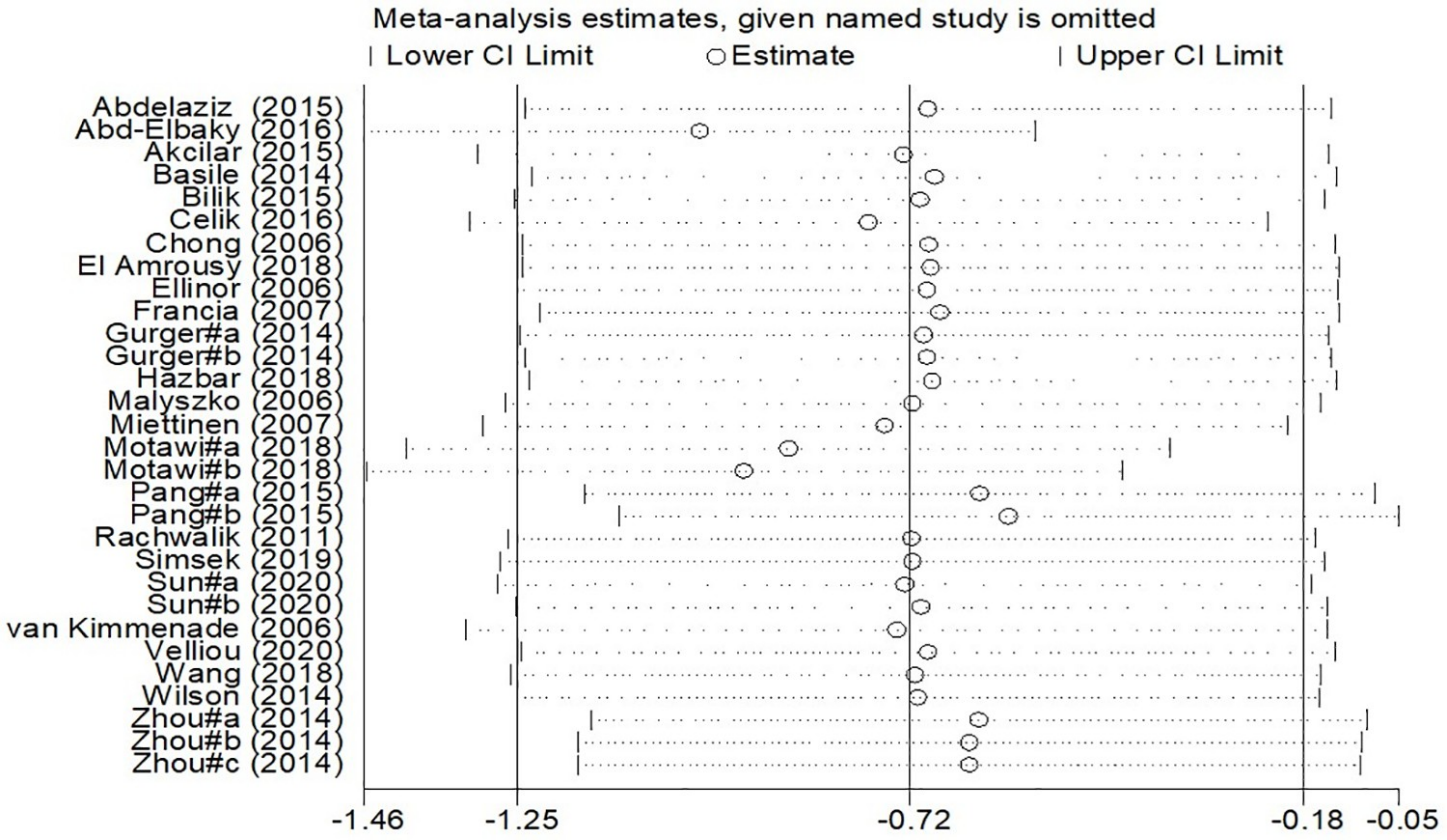
The sensitivity analysis results for standardized mean differences of circulating apelin levels between cases with CVDs and controls.

Sensitivity analyses showed that the exclusion of each study did not change the pooled SMD for apelin levels. In addition, the lower and higher pooled effects for our outcomes, after the one-by-one exclusion of the studies, are shown in Fig 3.

### Additional analyses

As observed in Table 2, the subgroup findings showed that the apelin levels in studies with a cohort or cross-sectional design, without medical comorbidities of diabetic/metabolic syndrome (MetS), plasma body fluid, patients with other disease, and studies conducted in Europe or Asia were statistically significant when compared to other strata. Meanwhile, the findings of univariate meta-regression analyses based on total sample size, publication year, and quality score did not indicate any significant associations with apelin levels (P ≥ 0.05 for all moderator variables).

**Table 2:** Subgroup analyses results.

Outcomes	Subgroups		No. studies	SMD	95% CI	I^2^ (%)
**Apelin**	Study design	Case-control	24	-0.66	-1.42, 0.10	97.7
		Cohort	4	-0.90	-1.72, -0.08	95.5
		Cross-sectional	2	-0.76	-1.05, -0.47	0.0
	Comorbidities of diabetes/MetS	With	14	-0.35	-1.23, 0.52	97.7
		Without	14	-1.20	-1.96, -0.43	96.7
		NR	2	-0.22	-1.96, -0.43	96.7
	Body fluid	Plasma	19	-1.57	(-2.13, -1.02)	96.4
		Serum	10	1.13	-0.31, 2.57	98.4
		NR	1	-1.38	-1.82, -0.95	-
	Heart diseases	CAD	12	-0.78	-1.85, 0.28	97.9
		Other disease	18	-0.65	-1.27, -0.03	96.8
	Continent	Africa	5	3.23	-0.45, 6.91	99.2
		Europe	13	-0.85	-1.24, -0.46	87.5
		America	2	-0.66	-1.93, 0.61	97.6
		Asia	10	-2.31	-3.14, -1.47	94.6

### Publication bias

Egger’s tests indicated no significant evidence of possible publication bias for apelin (Coef = -1.44, P = 0.556) levels. The visual-filled funnel plots and Egger’s test were used to evaluate the potential publication bias across the included studies as shown in Supp 2. b in S1 File.

## Discussion

CVDs are the leading global cause of death worldwide. As highly prevalent diseases, the early detection and cure of CVDs is a critical issue [4, 6]. In obesity, as one of the important risk factors of CVD, dysregulation of adipokines such as apelin originating from adipose tissue may result in the association between obesity and CVD [6, 41, 42]. To the best of the author’s knowledge, this is the first systematic review and meta-analysis to summarize available data regarding the circulating levels of apelin and its ratio to lipid profile in patients with CVDs. Findings obtained from the present meta-analyses revealed the significantly lower circulating levels of apelin among patients with CVD in comparison to those of the controls. Moreover, apelin was significantly lower in patients without diabetes/MetS. Furthermore, apelin/HDL-c, apelin/LDL-c, and apelin/TC ratios were associated with risk of CVD development.

Apelin, an endogenous peptide ligand of the 7-transmembrane G-protein, coupled with its receptor (APJ), is a strong inotrope and vasodilator [9, 43]. Apelin is secreted by white adipose tissue and its expression has also been identified in other tissues, including the heart, kidney, and endothelium [44–46]. Increased apelin expression has been found in cardiovascular tissues such as cardiomyocytes, endothelial cells, and vascular smooth muscle cells [47]. The apelin–APJ axis plays an important role in the cardiovascular system [48, 49]. Additionally, apelin has recently been implicated in the physiology of cardiovascular system in regard to cardiac contractility, endothelium-dependent vasodilation, and the reduction of vascular wall inflammation [9]. As a regulating peptide of cardiovascular, gastrointestinal, hypothalamus-hypophysis, and immune systems, apelin apears to regulate lipid metabolism and adiposity since it increases uncoupling protein 1 (Ucp1) mRNA levels (a marker of peripheral energy expenditure) in brown adipose tissue and Uco3 mRNA levels (a regulator of fatty acid export) in skeletal muscle [50, 51].

The findings obtained from the present study showed that the circulating levels of apelin in patients with CVD were significantly lower than those in the controls. Subgroup analyses based on medical comorbidities of diabetes/MetS revealed that apelin levels were significantly lower in CVD patients without diabetes and MetS than in the controls. Apelin synthesis in adipocytes is stimulated by insulin and its plasma levels are demonstrated to increase in relation to diabetes mellitus, IR, and hyperinsulinemia [52]. Furthermore, it appears that in obese patients, such as those with diabetes or CVD, in addition to obesity, the type of disease also impacts the levels of inflammatory or anti-inflammatory mediators [53]. Moreover, subgroup analyses based on the CVD type demonstrated that apelin levels were significantly lower in other CVD subgroups such as CHD, CHF, HF, AF, and AMI than in the controls. These findings account for the possible usefulness of apelin as an additional biomarker in the diagnosis of CVD in diabetic patients and in diagnosis of patients with CAD. The findings on new combined markers indicated a significant decrease in SMD of the apelin/HDL-c ratio, apelin/LDL-c ratio, and apelin/TC ratio. However, no significant differences were found in the SMD of the apelin/TG ratio in cases with CVDs compared to the control group. The effects of apelin on lipid metabolism have been described by previous studies; apelin was shown to inhibit lipolysis and increase the stability of lipid vacuoles by making them resistant to lipases. Accordingly, apelin is related to enhanced serum lipids and can be utilized as a predictor of premature atherosclerosis in T1DM patients [54, 55].

Despite a number of unresolved questions, it appears that apelin has significant therapeutic potential. The observed cardiovascular roles of apelin suggest that this peptide could be considered a potential candidate for addition to the standard therapy of CVDs.

It should be noted that this meta-analysis had strengths and limitations that must be taken into account. As the first comprehensive meta-analysis, the present study ascertained the association between apelin and CVDs. Publication bias was not detected in our study. Subgroup analysis was performed to identify the possible sources of heterogeneity. The findings of the present study may have important implications for future research into whether apelin and its receptor could have a clinical value in the prevention and treatment of the diseases. The obtained results should be interpreted with caution due to the high heterogeneity of the selected studies. Therefore, further large-scale studies are required to confirm these findings.

## Conclusion

The findings showed that the circulatory levels of apelin are significantly lower in CVD patients than in the controls. In addition, apelin levels were affected by the region and disease subtypes. Apelin could be considered an additional biomarker in the diagnosis of CVD in diabetic patients and patients with CAD. In addition, apelin/HDL-c, apelin/LDL-c, and apelin/TC ratios could be offered as diagnostic markers for CVD. However, as other factors, including smoking, dietary patterns, and coronary medication may also impact the plasma levels of apelin, further studies are required to define the risk factors affecting the levels of apelin and confirm these findings.

## Supporting information

S1 Checklist PRISMA_2020_checklist. (DOCX)

S1 File PubMed search strategy (Supp 2. a) and the visual-filled funnel plots to evaluate the potential publication bias (Supp 2. b). (DOCX)

## Abbreviations

ACS: Acute coronary syndrome
ADHF: Acute decompensated heart failure
AF: Atrial fibrillation
AMI: Acute myocardial infarction
BAV: Bicuspid aortic valve
BMI: Body mass index
CAD: Coronary artery disease
CAE: Coronary artery ectesia
CHD: Coronary heart disease
CHF: Congestive heart failure
CI: Confidence interval
CVD: Cardiovascular disease
ERAF: Early recurrence of atrial fibrillation
HD: Hemodialysis
HDL-C: High-density lipoprotein cholesterol
HF: Heart failure
HHD: Hypertensive heart disease
HT: hypertension
IDC: Idiopathic dilated cardiomyopathy
IHD: Ischemic heart disease
IR: Insulin resistance
LDL-C: Low-density lipoprotein cholesterol
MeSH: Medical Subject Heading
MetS: Metabolic syndrome
MI: Myocardial infarction
Non-STEMI: Non ST elevation myocardial infarction
NOS: Newcastle-Ottawa Quality Assessment Scale
NR: Not reported
PRISMA: Preferred Reporting Items for Systematic Reviews and Meta-analysis
PSVT: Paroxysmal supraventricular tachycardia
SAP: Stable angina pectoris
SD: Standard deviation
SMD: Standardized mean difference
STEMI: ST elevation myocardial infarction
T1DM: Type 1 diabetes mellitus
TC: Total cholesterol
TG: Triacylglycerol
UAP: Unstable angina pectoris
Ucp1: Uncoupling Protein 1
VLDL-C: Very low density lipoprotein cholesterol
WHO: World Health Organization

## References

[1] Akbari H, Asadikaram G, Jafari A, Nazari-Robati M, Ebrahimi G, Ebrahimi N, et al. Atorvastatin, losartan and captopril may upregulate IL-22 in hypertension and coronary artery disease; the role of gene polymorphism. Life sciences. 2018;207:525–31. doi: 10.1016/j.lfs.2018.07.005 29981321

[2] Akbari H, Asadikaram G, Aria H, Fooladi S, Vakili S, Masoumi M. Association of Klotho gene polymorphism with hypertension and coronary artery disease in an Iranian population. BMC cardiovascular disorders. 2018;18(1):1–7. 30547758 10.1186/s12872-018-0971-5 PMC6295088

[3] Akbari H, Asadikaram G, Vakili S, Masoumi M. Atorvastatin and losartan may upregulate renalase activity in hypertension but not coronary artery diseases: The role of gene polymorphism. Journal of cellular biochemistry. 2019;120(6):9159–71. doi: 10.1002/jcb.28191 30548657

[4] Stewart J, Manmathan G, Wilkinson P. Primary prevention of cardiovascular disease: A review of contemporary guidance and literature. JRSM cardiovascular disease. 2017;6:2048004016687211. doi: 10.1177/2048004016687211 28286646 PMC5331469

[5] Kheirmand Parizi M, Akbari H, Malek-Mohamadi M, Kheirmand Parizi M, Kakoei S. Association of salivary levels of immunoglobulin-a and amylase with oral-dental manifestations in patients with controlled and non-controlled type 2 diabetes. BMC oral health. 2019;19(1):175. Epub 2019/08/08. doi: 10.1186/s12903-019-0868-4 . 31387562 PMC6685263

[6] Motawi TM, Mahdy SG, El-Sawalhi MM, Ali EN, El-Telbany RFA. Serum levels of chemerin, apelin, vaspin, and omentin-1 in obese type 2 diabetic Egyptian patients with coronary artery stenosis. Canadian journal of physiology and pharmacology. 2018;96(1):38–44. doi: 10.1139/cjpp-2017-0272 28957639

[7] Szokodi I, Tavi P, Földes Gb, Voutilainen-Myllylä S, Ilves M, Tokola H, et al. Apelin, the novel endogenous ligand of the orphan receptor APJ, regulates cardiac contractility. Circulation research. 2002;91(5):434–40. doi: 10.1161/01.res.0000033522.37861.69 12215493

[8] Földes G, Horkay F, Szokodi I, Vuolteenaho O, Ilves M, Lindstedt KA, et al. Circulating and cardiac levels of apelin, the novel ligand of the orphan receptor APJ, in patients with heart failure. Biochem Biophys Res Commun. 2003;308(3):480–5. Epub 2003/08/14. doi: 10.1016/s0006-291x(03)01424-4 . 12914775

[9] Kadoglou NP, Lampropoulos S, Kapelouzou A, Gkontopoulos A, Theofilogiannakos EK, Fotiadis G, et al. Serum levels of apelin and ghrelin in patients with acute coronary syndromes and established coronary artery disease—KOZANI STUDY. Translational Research. 2010;155(5):238–46. doi: 10.1016/j.trsl.2010.01.004 20403579

[10] Bamba V. Update on screening, etiology, and treatment of dyslipidemia in children. The Journal of Clinical Endocrinology & Metabolism. 2014;99(9):3093–102. 24848708 10.1210/jc.2013-3860

[11] Page MJ, McKenzie JE, Bossuyt PM, Boutron I, Hoffmann TC, Mulrow CD, et al. The PRISMA 2020 statement: an updated guideline for reporting systematic reviews. Bmj. 2021;372. 10.1136/bmj.n71 PMC8005924 33782057

[12] Peterson J, Welch V, Losos M, Tugwell P. The Newcastle-Ottawa scale (NOS) for assessing the quality of nonrandomised studies in meta-analyses. Ottawa: Ottawa Hospital Research Institute. 2011:1–12.

[13] Alan Stuart K. Kendall’s advanced theory of statistics. London: Wiley. p; 1998.

[14] Elandt-Johnson RC, Johnson NL. Survival models and data analysis: John Wiley & Sons; 1980.

[15] Bilik MZ, Kaplan İ, Yıldız A, Akıl MA, Acet H, Yüksel M, et al. Apelin levels in isolated coronary artery ectasia. Korean circulation journal. 2015;45(5):386–90. doi: 10.4070/kcj.2015.45.5.386 26413106 PMC4580697

[16] Sun X, Zhang Y, Qi X, Wei L. Impact of apelin-13 on the development of coronary artery ectasia. Acta Cardiologica Sinica. 2020;36(3):216. doi: 10.6515/ACS.202005_36(3).20190901A 32425436 PMC7220970

[17] Higgins JP, Thompson SG. Quantifying heterogeneity in a meta-analysis. Statistics in medicine. 2002;21(11):1539–58. doi: 10.1002/sim.1186 12111919

[18] Abdelaziz AA, Daoud EM, Elmalky N, El-Hussiny MAB, Mohamed SA. Plasma apelin level after percutaneous coronary intervention. The Egyptian Heart Journal. 2015;67(1):63–8.

[19] Abd-Elbaky AE, Abo-ElMatty DM, Mesbah NM, Ibrahim SM. Omentin and apelin concentrations in relation to obesity, diabetes mellitus type two, and cardiovascular diseases in Egyptian population. International Journal of Diabetes in Developing Countries. 2016;36(1):52–8.

[20] Abdullah MH, Sahab KS. The Use of Plasma Apelin Alteration in Diagnosis of Atherosclerosis. Journal of Biochemical Technology. 2018;9(3):23.

[21] Akcılar R, Yümün G, Bayat Z, Donbaloğlu O, Erselcan K, Ece E, et al. Characterization of the apelin-1860T> C polymorphism in Turkish coronary artery disease patients and healthy individuals. International journal of physiology, pathophysiology and pharmacology. 2015;7(4):165. 27073592 PMC4788725

[22] Basile G, Crucitti A, Cucinotta M, Lacquaniti A, Catalano A, Loddo S, et al. Serum levels of Apelin-36 are decreased in older hospitalized patients with heart failure. European Geriatric Medicine. 2014;5(4):242–5.

[23] Celik Y, Yardan T, Baydin A, Demircan S. The role of NT-proBNP and Apelin in the assessment of right ventricular dysfunction in acute pulmonary embolism. Age (years). 2016;62:24–94. 26968282

[24] Chong KS, Gardner RS, Morton JJ, Ashley EA, McDonagh TA. Plasma concentrations of the novel peptide apelin are decreased in patients with chronic heart failure. European journal of heart failure. 2006;8(4):355–60. doi: 10.1016/j.ejheart.2005.10.007 16464638

[25] El Amrousy D, El-Mahdy H. Prognostic value of serum apelin level in children with heart failure secondary to congenital heart disease. Pediatric cardiology. 2018;39(6):1188–93. doi: 10.1007/s00246-018-1879-7 29632960

[26] Ellinor PT, Low AF, MacRae CA. Reduced apelin levels in lone atrial fibrillation. European heart journal. 2006;27(2):222–6. doi: 10.1093/eurheartj/ehi648 16278229

[27] Francia P, Salvati A, Balla C, De Paolis P, Pagannone E, Borro M, et al. Cardiac resynchronization therapy increases plasma levels of the endogenous inotrope apelin. European journal of heart failure. 2007;9(3):306–9. doi: 10.1016/j.ejheart.2006.06.005 16891152

[28] Gurger M, Celik A, Balin M, Gul E, Kobat MA, Bursali KB, et al. The association between apelin-12 levels and paroxysmal supraventricular tachycardia. Journal of Cardiovascular Medicine. 2014;15(8):642–6. doi: 10.2459/JCM.0000000000000010 24933193

[29] Malyszko J, Malyszko J, Pawlak K, Mysliwiec M. Visfatin and apelin, new adipocytokines, and their relation to endothelial function in patients with chronic renal failure. Advances in Medical Sciences (De Gruyter Open). 2008;53(1). 10.2478/v10039-008-0024-x 18635422

[30] Miettinen KH, Magga J, Vuolteenaho O, Vanninen EJ, Punnonen KR, Ylitalo K, et al. Utility of plasma apelin and other indices of cardiac dysfunction in the clinical assessment of patients with dilated cardiomyopathy. Regulatory peptides. 2007;140(3):178–84. doi: 10.1016/j.regpep.2006.12.004 17223209

[31] Pang H, Han B, Li Z, Fu Q. Identification of molecular markers in patients with hypertensive heart disease accompanied with coronary artery disease. Genet Mol Res. 2015;14(1):93–100. doi: 10.4238/2015.January.15.12 25729940

[32] Rachwalik M, Diakowska D, Kustrzycki W. Serum concentration of selected adipocytokines in patients with coronary artery disease suitable for surgical revascularization: a preliminary study. Kardiochirurgia i Torakochirurgia Polska. 2011;8(4):432–6.

[33] Şimşek EÇ, Yakar Tülüce S, Tülüce K, Emren SV, Çuhadar S, Nazlı C. The relationship between serum apelin levels and aortic dilatation in bicuspid aortic valve patients. Congenital Heart Disease. 2019;14(2):256–63. doi: 10.1111/chd.12718 30485657

[34] van Kimmenade RR, Januzzi JL, Ellinor PT, Sharma UC, Bakker JA, Low AF, et al. Utility of amino-terminal pro-brain natriuretic peptide, galectin-3, and apelin for the evaluation of patients with acute heart failure. Journal of the American College of Cardiology. 2006;48(6):1217–24. doi: 10.1016/j.jacc.2006.03.061 16979009

[35] Velliou M, Sanidas E, Papadopoulos D, Iliopoulos D, Mantzourani M, Toutouzas K, et al. Adipokines and Atrial Fibrillation. The Important Role of Apelin. Hellenic journal of cardiology: HJC = Hellenike kardiologike epitheorese. 2020. doi: 10.1016/j.hjc.2020.04.018 32387593

[36] Wang YZ, Fan J, Zhong B, Xu Q. Apelin: A novel prognostic predictor for atrial fibrillation recurrence after pulmonary vein isolation. Medicine. 2018;97(39). doi: 10.1097/MD.0000000000012580 30278567 PMC6181607

[37] Wilson V, Gupta N, Prabhakar P, Ramakrishnan L, Seth S, Maulik S. Effect of percutaneous transvenous mitral commissurotomy on plasma apelin level in mitral stenosis patients. J Clin Exp Cardiolog. 2014;5(283):2.

[38] Zhou Y, Wang Y, Qiao S. Apelin. International heart journal. 2014:13–234. 24806385 10.1536/ihj.13-234

[39] Małyszko J, Małyszko JS, Koźminski P, Myśliwiec M. Apelin and cardiac function in hemodialyzed patients: possible relations? American journal of nephrology. 2006;26(2):121–6. doi: 10.1159/000092122 16549903

[40] Zhou Y, Wang Y, Qiao S. Apelin: A potential marker of coronary artery stenosis and atherosclerotic plaque stability in ACS patients. International Heart Journal. 2014;55(3):204–12. doi: 10.1536/ihj.13-234 24806385

[41] Shah A, Mehta N, Reilly MP. Adipose inflammation, insulin resistance, and cardiovascular disease. JPEN Journal of parenteral and enteral nutrition. 2008;32(6):638–44. Epub 2008/11/01. doi: 10.1177/0148607108325251 . 18974244 PMC3088110

[42] Mattu HS, Randeva HS. Role of adipokines in cardiovascular disease. The Journal of endocrinology. 2013;216(1):T17. doi: 10.1530/JOE-12-0232 23160967

[43] Nowzari Z, Masoumi M, Nazari-Robati M, Akbari H, Shahrokhi N, Asadikaram G. Association of polymorphisms of leptin, leptin receptor and apelin receptor genes with susceptibility to coronary artery disease and hypertension. Life sciences. 2018;207:166–71. doi: 10.1016/j.lfs.2018.06.007 29883719

[44] Chandrasekaran B, Dar O, McDonagh T. The role of apelin in cardiovascular function and heart failure. European journal of heart failure. 2008;10(8):725–32. doi: 10.1016/j.ejheart.2008.06.002 18583184

[45] Hou X, Zeng H, He X, Chen JX. Sirt3 is essential for apelin-induced angiogenesis in post-myocardial infarction of diabetes. Journal of Cellular and Molecular Medicine. 2015;19(1):53–61. doi: 10.1111/jcmm.12453 25311234 PMC4288349

[46] Li L, Zeng H, Hou X, He X, Chen J-X. Myocardial injection of apelin-overexpressing bone marrow cells improves cardiac repair via upregulation of Sirt3 after myocardial infarction. PloS one. 2013;8(9):e71041. doi: 10.1371/journal.pone.0071041 24039710 PMC3765164

[47] Liu H-T, Chen M, Yu J, Li W-J, Tao L, Li Y, et al. Serum apelin level predicts the major adverse cardiac events in patients with ST elevation myocardial infarction receiving percutaneous coronary intervention. Medicine. 2015;94(4). doi: 10.1097/MD.0000000000000449 25634182 PMC4602953

[48] Antushevich H, Wójcik M. Apelin in disease. Clinica chimica acta. 2018;483:241–8. 10.1016/j.cca.2018.05.012 29750964

[49] Arababadi MK, Asadikaram P, Asadikaram G. APLN/APJ pathway: The key regulator of macrophage functions. Life sciences. 2019;232:116645. doi: 10.1016/j.lfs.2019.116645 31299236

[50] Sabry RN, El Wakeel MA, El-Kassas GM, Amer AF, El Batal WH, El-Zayat SR, et al. Serum apelin: a new marker of early atherosclerosis in children with type 1 diabetes mellitus. Open access Macedonian journal of medical sciences. 2018;6(4):613. doi: 10.3889/oamjms.2018.144 29731925 PMC5927488

[51] Lago F, Gómez R, Gómez-Reino JJ, Dieguez C, Gualillo O. Adipokines as novel modulators of lipid metabolism. Trends in biochemical sciences. 2009;34(10):500–10. doi: 10.1016/j.tibs.2009.06.008 19729309

[52] Iwanaga Y, Kihara Y, Takenaka H, Kita T. Down-regulation of cardiac apelin system in hypertrophied and failing hearts: possible role of angiotensin II–angiotensin type 1 receptor system. Journal of molecular and cellular cardiology. 2006;41(5):798–806. doi: 10.1016/j.yjmcc.2006.07.004 16919293

[53] Japp A, Cruden N, Barnes G, Van Gemeren N, Mathews J, Adamson J, et al. Acute cardiovascular effects of apelin in humans. Circulation. 2010;121(16):1818–27. doi: 10.1161/CIRCULATIONAHA.109.911339 20385929

[54] Yue P, Jin H, Xu S, Aillaud M, Deng AC, Azuma J, et al. Apelin decreases lipolysis via Gq, Gi, and AMPK-dependent mechanisms. Endocrinology. 2011;152(1):59–68. doi: 10.1210/en.2010-0576 21047945 PMC3033059

[55] Than A, Cheng Y, Foh L-C, Leow MK-S, Lim SC, Chuah YJ, et al. Apelin inhibits adipogenesis and lipolysis through distinct molecular pathways. Molecular and cellular endocrinology. 2012;362(1–2):227–41. doi: 10.1016/j.mce.2012.07.002 22842084

