# Supplementary material for: Apelin and its ratio to lipid factors are associated with cardiovascular diseases: A systematic review and meta-analysis
Source: PLoS One. 2022 Aug 1;17(8):e0271899. doi: 10.1371/journal.pone.0271899 (PMC9342781; doi:10.1371/journal.pone.0271899)
Supplement: S1 File — (DOCX) [file pone.0271899.s002.docx]

**Supp 2. a: Search strategy**

**Pubmed 407**

(Apelin[mh] OR Apelin[tiab]) AND ("coronary heart disease"[tiab] OR "Coronary Artery Disease"[Mesh] OR "Coronary Artery Disease"[tiab] OR "Artery Disease"[tiab] OR "Coronary Arteriosclerosis"[tiab] OR "Coronary Atherosclerosis"[tiab] OR "Atherosclerosis"[tiab] OR "CAD"[tiab] OR "Myocardial Ischemia"[Mesh] OR "Myocardial Ischemia"[tiab] OR "Ischemia"[tiab] OR "Myocardial"[tiab] OR "Ischemic Heart Disease"[tiab] OR "Heart Disease"[tiab] OR "Ischemic"[tiab] OR "Acute Coronary Syndrome"[Mesh] OR "Acute Coronary Syndrome"[tiab] OR "Coronary Syndrome"[tiab] OR "Acute Coronary"[tiab] OR "ACS"[tiab] OR "Angina, Stable"[Mesh] OR "Angina"[tiab] OR "Stable Angina"[tiab] OR "Chronic Stable Angina"[tiab] OR "Angina, Unstable"[Mesh] OR "Unstable Angina"[tiab] OR "Coronary Disease"[Mesh] OR "Coronary Disease"[tiab] OR "Coronary"[tiab] OR "Coronary Stenosis"[Mesh] OR "Coronary Stenosis"[tiab] OR "Stenosis"[tiab] OR "Myocardial Infarction"[Mesh] OR "Myocardial Infarction"[tiab] OR "Infarction"[tiab] OR "Cardiovascular Stroke"[tiab] OR "Cardiovascular Stroke"[tiab] OR "Stroke"[tiab] OR "Myocardial Infarct"[tiab] OR "Infarct"[tiab] OR "Heart Attack"[tiab] OR "Heart Attack"[tiab] OR "MI"[tiab] OR "Non-ST Elevated Myocardial Infarction"[Mesh] OR "Non ST Elevated Myocardial Infarction"[tiab] OR "NSTEMI"[tiab] OR "Non-ST-Elevation Myocardial Infarction"[tiab] OR "Non-ST-Elevation Myocardial"[tiab] OR "Non-ST-Elevation"[tiab] OR "Non ST Elevation Myocardial Infarction"[tiab] OR "ST Elevation Myocardial Infarction"[Mesh] OR "ST Segment Elevation Myocardial Infarction"[tiab] OR "ST Elevated Myocardial Infarction"[tiab] OR "STEMI"[tiab] OR "Heart Disease"[tiab] OR "Coronary Occlusion"[tiab] OR Stenosis[tiab])

**Filters applied: English, Humans.**

**Supp 2. b: Funnel plot**
